# Bioprosthetic mitral valve thrombosis

**DOI:** 10.1007/s12471-022-01659-x

**Published:** 2022-02-04

**Authors:** S. Bouwmeester, M. el Farissi, P. Houthuizen

**Affiliations:** Department of Cardiology, Catharina Hospital Eindhoven, Eindhoven, The Netherlands

An 80-year-old woman presented with an ischaemic stroke one year after an uneventful bioprosthetic mitral valve replacement. Transthoracic echocardiography was of suboptimal image quality; however, Doppler interrogation of the mitral valve revealed a markedly elevated transvalvular gradient (Fig. 1a). Transoesophageal echocardiography showed a large, mobile mass, which was attached to the mitral bioprosthesis and mimicked a ‘thumbs-up sign’ (Fig. 1b; see also Video 1 in the Electronic Supplementary Material). As the patient was afebrile and both blood cultures and ^18^FDG PET/CT imaging were negative, bioprosthetic valve thrombosis (BPVT) was considered a more likely diagnosis than endocarditis. Patient was declined for surgery because of the high operative risk. Alternatively, treatment with a warfarin derivate was initiated. Follow-up echocardiography showed a gradual decrease of the echogenic structure size over time (Fig. 1c, d; see also Videos 2 and 3 in the Electronic Supplementary Material), with normalization of the transvalvular gradient.

**Fig. 1:**
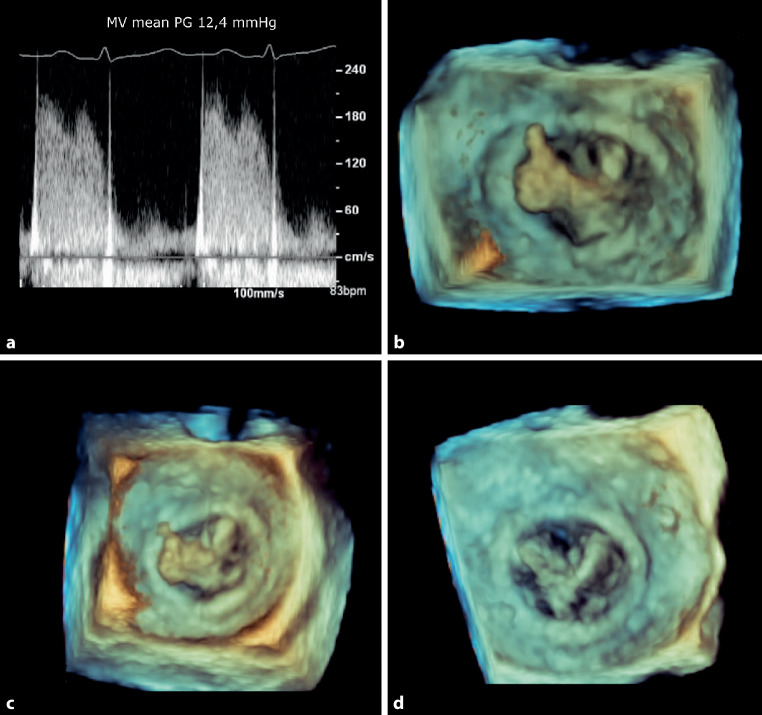
Bioprosthetic valve thrombosis **a** Elevated transvalvular gradient. **b** Echogenic mass attached to mitral bioprosthesis. **c,** **d** Gradual decrease of echogenic mass under oral anticoagulant therapy

Clinicians should be aware of BPVT, especially in a patient who presents with a thromboembolic event. Prompt echocardiographic evaluation is essential for the diagnosis of BPVT. Symptomatic BPVT is rare, occurring in < 1% of patients undergoing surgical valve implantation. Symptomatic BPVT with a large thrombus (≥ 1.0 cm) requires urgent intervention. In general, surgery is the preferred treatment for symptomatic BPVT. However, fibrinolysis or oral anticoagulants should be considered in high–surgical risk patients.

## Supplementary Information

**Video 1** Echogenic mass attached to mitral bioprosthesis

**Video 2** Gradual decrease of echogenic mass under oral anticoagulant therapy

**Video 3** Gradual decrease of echogenic mass under oral anticoagulant therapy

